# Systematic review of universal resilience interventions targeting child and adolescent mental health in the school setting: review protocol

**DOI:** 10.1186/s13643-015-0172-6

**Published:** 2015-12-29

**Authors:** Julia Dray, Jenny Bowman, Luke Wolfenden, Elizabeth Campbell, Megan Freund, Rebecca Hodder, John Wiggers

**Affiliations:** Hunter New England Population Health, Hunter New England Local Health District, Locked Bag 10, Wallsend, NSW 2287 Australia; The University of Newcastle, Callaghan, NSW 2308 Australia; Hunter Medical Research Institute, Locked Bag 1, Hunter Region Mc, NSW 2310 Australia

**Keywords:** Mental health, Adolescent, Child, Resilience, Intervention, School, Protective factors

## Abstract

**Background:**

The mental health of children and adolescents is a key area of health concern internationally. Previous empirical studies suggest that resilience may act as a protective mechanism towards the development of mental health problems. Resilience refers to the ability to employ a collection of protective factors to return to or maintain positive mental health following disadvantage or adversity. Schools represent a potential setting within which protective factors of all children and adolescents may be fostered through resilience-focussed interventions. Despite this potential, limited research has investigated the effectiveness of universal school-based resilience-focussed interventions on mental health outcomes in children and adolescents. The objective of the present review is to assess the effects of universal school-based resilience-focussed interventions, relative to a comparison group, on mental health outcomes in children and adolescents.

**Methods/design:**

Eligible studies will be randomised (including cluster-randomised) controlled trials of universal interventions explicitly described as resilience-focussed or comprising strategies to strengthen a minimum of three internal protective factors, targeting children aged 5 to 18 years, implemented within schools, and reporting a mental health outcome. Screening for studies will be conducted across six electronic databases: MEDLINE, PsycINFO, Educational Resources Information Center (ERIC), Excerpta Medica database (EMBASE), Cumulative Index to Nursing and Allied Health Literature (CINAHL), and the Cochrane Central Register of Controlled Trials (CENTRAL). Two reviewers will retrieve eligible articles, assess risk of bias, and extract data. Where studies are sufficiently homogenous and reported outcomes are amenable for pooled synthesis, meta-analysis will be performed. Narrative description will be used to synthesise trial outcome data where data cannot be combined or heterogeneity exists.

**Discussion:**

This review will aid in building an evidence base for the effectiveness of universal school-based resilience-focussed interventions and in doing so provide an opportunity to better inform the development of interventions to potentially prevent mental health problems in child and adolescent populations.

**Systematic review registration:**

PROSPERO CRD42015025908

## Background

The mental health of children and adolescents has been identified internationally [1–3] as a priority area for additional research and government action. Worldwide, the prevalence of mental health problems in children and adolescents is reported to be between 10 and 20 % [4]. Additionally, a higher prevalence of mental health problems is generally reported for particular sub-populations of young people, including those of lower socio-economic status [5–7], belonging to minority ethnic groups [8, 9], and young people living in more rural or remote areas [10]. Mental health problems in adolescents have been shown to increase the risk of negative outcomes such as disability, loss of productivity and contribution to the community, lower educational achievement, higher likelihood of engagement in health risk behaviours, and higher rates of self-harm and suicide [11, 12]. Across international literature, the mental health problems reported to be most prevalent and have the greatest impact on children and adolescents are depression, anxiety, disruptive behaviour disorders, attention deficit hyperactivity disorder, and substance use disorders [13–20].

Previous research provides support for the premise that resilience may be protective against the development of mental health problems [21–23]. Despite recent growth in the field of resilience research, the terminology used to describe the concept of resilience, and the qualities that constitute the process or outcome, continue to vary greatly [24], and there is no universally employed operationalization [25]. However, it is suggested by prominent researchers that ‘resilience’ is a collection of protective factors (resources and assets) that when strengthened and employed by an individual during times of disadvantage or adversity promote desirable outcomes such as the maintenance of or return to positive mental health or the prevention of negative mental health outcomes [25, 26]. Studies have found high levels of protective factors, such as personal and social competence, perceived level of family cohesion, and social resources, to be associated with positive mental health outcomes such as reduced symptoms or levels of depression [21–23], anxiety, stress, and obsessive compulsive disorder in adolescents [22].

Resilience-focussed interventions seek to strengthen protective factors and in doing so foster the development of coping mechanisms and positive mental health [27]. Protective factors have been defined as factors that alter, in a positive direction, the manner in which a person responds to disadvantage or adversity [28, 29], and are often perceived to incorporate both internal factors that reside within the individual (such as self-efficacy, coping skills, and effective problem solving) and external factors that include characteristics of the wider social environment (such as family and peer relationships, and support and meaningful participation within home, school, or community environments) [23, 27, 30, 31]. Universal interventions are those that target whole populations or groups of adolescents not identified as having or being at-risk of mental health problems [32]. Thus, universal interventions are a potential platform for addressing key health issues such as mental health problems in adolescents at a population level. Schools offer an opportune setting for the implementation of universal resilience-focussed interventions as they provide centralised access to large numbers of children and adolescents over long periods of time, at a critical time in child development, and have existing resources and infrastructure to support child development [33, 34]. A number of universal resilience-focussed interventions targeting mental health outcomes such as the overall improvement of mental health or reduction in the prevalence of mental health problems in children and adolescents have been conducted and reported to have positive effects [23, 35].

Two recent reviews have been conducted on the effectiveness of resilience-focussed interventions on various outcomes in adults [36, 37]. The first, a review by Macedo et al., included randomised, non-randomised, and open-ended studies of interventions aimed at promoting resilience. The review included 13 studies and found most interventions to have reported an increase in resilience variables for non-clinical samples of adults [36]. No mental health outcomes were reported in the review. The second review by Leppin et al. included only randomised controlled trials. The review included 25 trials and found a modest but consistent effect of resiliency training programs on enhancing resilience and in improving mental health outcomes including stress and depression [37].

However, existing systematic reviews of resilience-focussed interventions in children and adolescents are limited and have not synthesised the body of evidence regarding the impact of universal school-based resilience-focussed interventions on mental health outcomes. A meta-analysis of 17 controlled evaluations of one cognitive behavioural intervention in schools targeting resilience factors in children and adolescents, the PENN Resiliency Program, indicated positive results in terms of reduction of depressive symptoms for both universal (6 trials) and targeted (11 trials) application of the intervention [38]. However, as stated, the review focused on only one particular intervention on the single outcome of depressive symptoms and thus did not synthesise evidence on the impact of multiple resilience-focussed interventions on mental health outcomes in children and adolescents. Additionally, a more recent review by Brownlee et al. [39] aimed to identify outcome literature relating to strength-based and resilience-based interventions relevant to children and adolescents and examine the extent to which such trials utilised controlled empirical methodology. Eleven eligible trials were identified, with three conducted using rigorous experimental methods and eight using moderate or weak level experimental methods [39]. The review concluded that the studies provided preliminary support for the efficacy of strength and resilience-based interventions. However, the review was not limited to studies in a school setting, did not report the effects by this setting, did not focus on universal interventions, indicated no restriction on reported comparator or outcome, and therefore did not specifically report mental health outcomes [39].

Given the potential for such interventions and the limitations of previous systematic reviews in this area, a review of the effectiveness of universal school-based resilience-focussed interventions on mental health outcomes in children and adolescents is warranted.

### Objective

The aim of the review is to assess the effects of universal (interventions targeted to the whole student population or entire groups of students not identified as having or being at-risk of mental health problems) school-based resilience-focussed interventions relative to a comparison group on mental health outcomes in children and adolescents aged 5 to 18 years.

## Methods

### Eligibility criteria

#### Study characteristics

##### Participants

Studies will be eligible for inclusion if they report on children or adolescents aged 5 to 18 years of age attending school. Studies including participants outside this age range will be included only if the mean age of participants at the time of enrolment in the study is between 5 and 18 years. Studies that select participants on the criteria of an existing self-report or diagnosed mental illness or cognitive or developmental disability will be excluded.

##### Study design

Eligible trials will include randomised controlled trials (RCTs), including cluster-randomised controlled trials (CRCTs), that compare a school-based resilience-focussed intervention program with:

1. an alternative intervention; or,
2. a control or comparison group. Control or comparison groups may include comparator groups that receive no intervention, usual practice, or attention only [40].

##### Setting

For a study to be eligible for inclusion, the intervention, or at least a substantive part of the intervention content, must be school-based. Studies will be considered as school-based if the intervention is demonstrated to be an extension of school participation. Intervention content must be received by the children and/or adolescents. Included schools will be schools attended by children and/or adolescents aged between 5 and 18 years. International terminology to describe school level varies; thus, included school settings may include, but are not limited to, schools described as infant, middle, elementary, primary, secondary, and high schools. Studies conducted at pre-schools or tertiary institutions such as college or university will be excluded.

##### Primary outcomes

Studies will be included if they report a measure of prevalence or extent of occurrence of child or adolescent mental health problems. Mental health data collected via various methods will be included, for example observational data; phone, online, or face-to-face self-report data; and secondary report e.g. by teachers or parents and guardians. Measures of mental health outcomes will not be required to be validated. Studies with outcomes of symptomology only will be excluded, for example sleeplessness and fatigue associated with depression. Studies where follow-up data collection is only conducted once participants reach 18 years of age or older will additionally be excluded.

##### Intervention

Interventions of included trials must:

- ▪ be universal (e.g. offered to whole-school, whole-year, or whole class) [32]. As such, studies targeted to the whole student population or entire groups of students not identified as having or being at-risk of mental health problems will be included [32]; and,
- ▪ explicitly state that the intervention is resilience-focussed. Under this criteria, resilience must be referred to in the title or key sentences defining the nature of the intervention for a study to be included. The rationale for this criteria is the acknowledgement that throughout the resilience research field, a high level of inconsistency in the operationalisation of resilience is evident, with no one definition of the concept or combination of protective factors accepted as the most accurate or robust [25, 41, 42]. Therefore, in order for the present review to be inclusive, varying approaches to resilience must be equally considered; or
- ▪ interventions must address at least three internal protective factors. Whilst variation exists in the operationalisation of resilience, it is generally accepted that the nature of resilience is multifactorial [25], suggesting the importance of strengthening multiple factors. This criteria is based on existing literature and the minimum number of internal protective factors reported as targeted in established resilience-focused interventions targeting mental health outcomes in children and adolescents [23, 31, 43–45].

Resilience-focussed interventions take many forms and therefore may vary by mode of delivery (e.g. school staff, research staff, external providers, or student facilitators), range in activity type (e.g. classroom-based exercises to build protective factors, presentations, special assemblies), or format of intervention (e.g. face-to-face curriculum-based or internet-based). There will be no exclusion based on duration of intervention, length of follow-up, mode of intervention delivery, or format of intervention.

In the event that a study includes both resilience-focussed and additional non-resilience-focussed content, details of intervention elements will be extracted and a narrative description will be used to synthesise this information.

##### Exclusion criteria

The following exclusion criteria will apply:

- ▪ Studies that report on interventions that are selective (studies of students considered at-risk of developing mental health problems), indicated (aimed at students with significant symptoms but that have not yet been diagnosed with a mental health problem), or treatment interventions (aimed at students with a current diagnosed mental disorder) [32] will be excluded.
- ▪ Interventions must be an extension of school participation. Therefore, interventions that only use schools for recruitment purposes will be excluded.

#### Publication characteristics

There will be no exclusion on the basis of study country; however, only studies published in English will be included. Studies published in the last 20 years will be eligible for inclusion.

### Information sources

#### Electronic databases

The following range of electronic databases will be searched: MEDLINE, PsycINFO, Educational Resources Information Center (ERIC), Excerpta Medica database (EMBASE), Cumulative Index to Nursing and Allied Health Literature (CINAHL), and the Cochrane Central Register of Controlled Trials (CENTRAL, The Cochrane Library).

#### Other sources

Hand searches for eligible studies will be conducted of reference lists of included studies: the three most relevant past reviews, the first 200 articles from Google Scholar, and volumes from the past five years of three relevant journals in the field (Journal of Child Psychology and Psychiatry, Journal of the American Academy of Child and Adolescent Psychiatry, Advances in School Mental Health Promotion).

### Search strategy

The search strategy will include terms for population, intervention, outcome, and study design (see Appendix 1 for the search strategy for MEDLINE). A published search filter will be used for the section of the search strategy pertaining to study design (randomised controlled trials; Cochrane 2008 Highly Sensitive Search Strategy) [46]. The search strategy will be modified where necessary to search individual electronic databases.

### Study records

#### Data management

The program EndNote will be used to remove duplicates, to assist in obtaining full text papers, and to store and manage records throughout the review. RevMan software will be used for pooling of trial data and meta-analyses.

#### Study selection process

Duplicate articles will be removed. Titles and abstracts of studies retrieved via the above search strategy will be independently screened by two reviewers to determine eligibility based on the predefined inclusion criteria, and articles that do not meet inclusion criteria will be excluded. Full-text papers of potentially eligible studies will be obtained and independently assessed by two reviewers against study inclusion criteria. Disagreements between reviewers regarding study eligibility will be resolved via consensus or if required by a third reviewer. Where insufficient study details exist, corresponding authors will be contacted for further details in order to determine study eligibility. In cases where further study details remain unavailable, studies will be deemed ineligible. Review authors will not be blinded to author name, author study institution, or journal title.

### Data extraction

The authors will extract and include data for every mental health outcome for each included study. Data will be independently extracted from eligible studies by two review authors. A data extraction form will be pre-piloted and used by the two authors during data extraction for assessment of study quality and evidence synthesis. Disagreements regarding data extraction will first be attempted to be resolved through discussion and consensus between the two authors.A third author will review any studies for which discrepancies remain unresolved. Where insufficient study data exists, corresponding authors will be contacted for clarification. One review author will transcribe data from eligible studies into RevMan software using data extraction forms, and the second review author will check this process.

### Data items

Information extracted from eligible studies will include the following: author(s) and year of publication, year(s) of study, country, study design, study population and participant demographics (including age and gender), study setting (to confirm school-based), intervention and comparison group conditions (including number of conditions, the protective factors targeted, and intervention duration and intensity), follow-up data collection points, trial outcomes and results (including consent, participation and attrition rate(s), sample size, results of relevant mental health outcomes, and intraclass correlation if relevant), measurement tool, details of intervention fidelity, and study funding and/or other sources of conflicts of interest and information required for assessment of potential study bias (see below).

### Assessment of risk of bias

As outlined in the Cochrane Handbook for Systematic Reviews of Interventions, risk of bias in included studies will be assessed independently by two review authors against the following qualities: selection bias (random sequence generation and allocation concealment), performance bias (blinding of participants and personnel), detection bias (blinding of outcome assessment), attrition bias (incomplete outcome data), reporting bias (selective reporting), and any other potential sources of bias [47].

Resolution of disagreements on risk of bias will first be attempted through discussion and consensus of the two review authors. In the event that consensus cannot be reached, a third reviewer will be consulted.

### Data analysis

Data from included studies will be extracted and intervention effects assessed on measures of comparable mental health outcomes. For studies reporting follow-up assessment, data will be extracted and reported according to short-term (<12 months) and long-term (>12 months) effects. For studies reporting multiple follow-up assessments, data from the final trial endpoint within the eligible participant age range (i.e. 5 to 18 years) will be used. Where studies are sufficiently homogenous, meta-analysis will be performed using a random effects model. Different outcomes will not be combined in pooled synthesis. Only measures of the same outcome will be pooled using a random effects model. Separate meta-analysis will be conducted for each outcome. Meta-analysis will, however, be contingent on the availability of appropriate data and following assessment and consideration of heterogeneity (described below under the ‘Data synthesis and analysis’ section). Binary outcomes will be pooled and effect estimates reported as relative risks. Continuous outcomes will be pooled and reported as a mean difference where consistent outcome measures are employed across studies or a standardised mean difference where different measures are used to report a comparable outcome. Variability in point estimates will be described using 95 % confidence intervals. If possible, additional sub-group analysis by age and gender will be performed. Attempts will be made to contact study authors to obtain missing data. Trials with missing data will be identified in the risk of bias assessment tables. The outcomes of trials unable to be included in meta-analysis due to missing data will be reported narratively.

#### Data synthesis and analysis

Heterogeneity will be assessed via visual inspection of forest plots and consideration of the *I*^2^ statistic (*I*^2^ of 75 to 100 % indicating considerable heterogeneity) [47]. Where heterogeneity exists, the sources of heterogeneity will be investigated through sub-group analysis on participant, design, outcome, and study quality characteristics. Narrative description will be used to synthesise trial outcome data where data cannot be combined or significant heterogeneity exists.

#### Issues of clustering

Due to the nature of the review (focus on school-based studies), it is expected that cluster-randomised control trials will be identified and potentially included. In such trials, where no adjustment has been made for the effect of clustering, intraclass correlations will be requested from corresponding authors, or where not available, estimates will be obtained from similar studies (e.g. school-based studies reporting similar school, student, gender and scholastic year characteristics) and combined using a generic inverse variance approach.

### Assessment of reporting bias

Funnel plots will be used to assess possible reporting bias in included studies.

### Confidence in cumulative evidence

The strength of the body of evidence and therefore the confidence in cumulative evidence will be assessed using the GRADE approach developed by the Grades of Recommendation, Assessment, Development and Evaluation Working Group [48–50]. This approach includes assessment of each individual outcome per trial across five key areas: risk of bias within included studies (methodological quality), directness of evidence (relevance to the review question), heterogeneity (inconsistency), precision of effect estimates, and risk of publication bias [50].

### Ethics and dissemination

No ethics approval was necessary for the present systematic review and therefore was not obtained. Dissemination of review findings is planned to occur through publication of the final review manuscript and conference presentations.

## Discussion

This systematic review will provide an evidence base for the effectiveness of school-based resilience-focussed interventions on mental health outcomes in children and adolescents. Such an evidence base provides an opportunity to better inform the development of interventions which may enable advantageous outcomes such as the maintenance or return to positive mental health, or prevention or reduction of mental health problems, in children and adolescents. Thus, this review will be of value to researchers, policy makers, and members of the community with an interest in supporting the mental health, well-being, resilience, and therefore overall positive life trajectories of children and adolescents.

## Appendix 1

Database(s): Ovid MEDLINE® 1946 to present with daily update

Search date: December 8–10, 2015

**Table 1:** Search strategy

#	Searches	Results
1	mental health.mp. or Mental Health/	119202
2	Stress, Psychological/ or psychological distress.mp.	101936
3	mental hygiene.mp.	2825
4	psychological status.mp.	1787
5	(psychological adj (wellbeing or well being or health)).mp.	8043
6	psychopathology.mp. or Psychopathology/	25328
7	(psychological* adj (stress* or adapt*)).mp.	6224
8	mental* ill*.mp. or Mentally Ill Persons/	31696
9	(emotional* adj (distress* or health or wellbeing or well being)).mp.	7875
10	exp Mental Disorders/ or mental disorder*.mp.	1035426
11	1 or 2 or 3 or 4 or 5 or 6 or 7 or 8 or 9 or 10	1186238
12	School Health Services/ or school*.mp.	229734
13	school based.mp.	7915
14	classroom.mp.	8682
15	(school* adj3 (intervention* or program* or course* or polic* or practice* or curricul* or environment*)).mp.	17470
16	schoolchild*.mp.	10807
17	12 or 13 or 14 or 15 or 16	234430
18	resilien*.mp. or Psychological Resilience/	12613
19	(factor* adj (protect* or promoti* or external or internal or environment*)).mp.	2995
20	(strength* adj (based or focused)).mp.	507
21	(development adj3 (adolescen* or youth or child* or positive)).mp.	64536
22	(coping or adaptability or life skill* or lifeskill* or social skill*).mp.	44408
23	(competence adj3 (emotional or behavio?ral or social or cognitive)).mp.	2748
24	psychological adaptation.mp. or Adaptation, Psychological/	80080
25	(positive psychology or psychosocial or psycho social or positive education).mp.	66410
26	18 or 19 or 20 or 21 or 22 or 23 or 24 or 25	235055
27	randomized controlled trial.pt.	418071
28	controlled clinical trial.pt.	92294
29	random*.tw.	730987
30	placebo.tw.	164067
31	clinical trials as topic.sh.	180293
32	trial.tw.	382569
33	27 or 28 or 29 or 30 or 31 or 32	1223015
34	11 and 17 and 26 and 33	845
35	limit 34 to yr = "1995 -Current"	764
36	(animals not (humans and animals)).sh.	4062702
37	35 not 36	764

## Abbreviations

RCT: randomised controlled trial
CRCT: cluster-randomised controlled trial
ERIC: Educational Resources Information Center
EMBASE: Excerpta Medica database
CINAHL: Cumulative Index to Nursing and Allied Health Literature
CENTRAL: Cochrane Central Register of Controlled Trials
GRADE: Grading of Recommendations Assessment, Development and Evaluation
